# Neuritis and Neuralgia

**Published:** 1895-03-02

**Authors:** 


### Neuritis and Neuralgia.

The man who invents a new disease is apt to be regarded at best with respectful aversion. He is thought to have added one more to the woes of mankind, whereas, in truth, he has but differentiated. Single names have covered, and still cover, many morbid states which, with increased knowledge, become separated and placed under different categories, and thus it has happened that neuralgia, a term which has so long sheltered all sorts of pains of uncertain origin, has been of late years gradually shorn of a good deal of its importance, organic causes having been in many cases found for pains which formerly were called neuralgia, and among these organic causes stands pre-eminent that morbid change which is spoken of as neuritis.

Whatever may be the essence of the early stages of neuritis, at the stage at which its pathology can be investigated, it is a mixed condition of inflammation and degeneration of the nerves, the first predominating in the fibrous coverings, the latter in the nerve tissues themselves. Dr. Cagney, in a paper read before the Islington Medical Society, has recently shown how many of the signs which have usually been looked upon as indicative of the neuralgic origin of pain, occur also in cases of neuritis. Among these, localisation in conformity with the distribution of peripheral nerves is markedly characteristic of the latter condition. Tender points, *points douloureux*, have been stated on high authority to be diagnostic of neuralgia, and have been very generally recognised as such. They have been shown to occur principally where branches from deeper nerves become superficial, and much stress has been laid upon them. But there is no doubt that in neuritis also the occurrence of these tender points is constant.

Periodicity again is a matter which is always inquired about, and is held to indicate nerve origin. But in neuritis pain may be periodic, and Dr. Cagney tells us that the best example of periodicity he ever saw was in the case of syphilitic neuritis. In fact, it would appear not improbable that the idea that periodicity means nerve is a relic or tradition from the times when ague was much more prevalent than it is now, and was the common cause of what was called neuralgia, a disease which it tainted with its own tendency to recur at stated periods. We know now, however, that malaria is a common cause of neuritis, as well as of neuralgia, and as a distinguishing factor, periodicity, falls to the ground. In fact now, as before, the term neuralgia remains the refuge for those cases which appear independent of gross lesion, and with greater diagnostic skill gross lesions are more often found, and the residual neuralgias grow less. Hence the importance in every case of a search for the associated signs which point to a neuritis. These consist chiefly of various disturbances of the other besides the mere pain-bearing functions of the nerve. Thus we may have weakness, paresis, or flabbiness of certain muscles, and often these muscles are tender. There may be a general tenderness, a superficial as well as a deep hypnosthesia, and, more important still, there may be varying degrees of anasthesia, corresponding to the distribution of certain nerves, and on careful search the nerves themselves may be found to be thickened. Then trophic changes must be looked for, such as herpetic eruptions, ridged nails, glossy skin, various joint affections, and errors of secretion.

Nevertheless, there will always be found a residuum of cases which must still be called neuralgia. The absolutely intermittent cases, in which the intensest pain alternates with long and uncertain periods of apparently normal health, or in which the most violent paroxysms are at once relieved by a purgative or an opiate and leave no trace, are probably independent of gross lesion. In fact, if we look upon neuralgia as being in essence an abnormal irritability of the sensory centres, it is not difficult to believe that this irritability may be set up by certain conditions of the blood, certain forms of toxæmia which, as we see clinically, are often capable of being removed or lessened by medicinal treatment. It must not, however, be forgotten that many of these morbid states are quite as capable of setting up a neuritis in the periphery as an irritability in the centre.

Thus anæmia, although a common enough cause of neuralgia, is also, possibly *viâ* the fœcal sapræmia with which it is so often associated, frequently the cause of a neuritis.

Rheumatism, again, is a cause of neuritis, while what is often spoken of as muscular rheumatism is in many cases really a neuritis due to alcohol. Gout and lead, either together or separate, cause neuritis; the one more commonly showing itself as pain, the other as paralysis. Malaria may be a cause of neuritis as well as of neuralgia, and the same may be said both of syphilis and diabetes. We are driven back then in our attempts at differentiation to a careful search for the associated symptoms which show degeneration and altered function of the nerves. In the chronic, persistent, inveterate cases of so-called neuralgia, a diagnosis as to the presence or absence of neuritis is of much interest and importance, while in the more ephemeral cases which come and go under the influence of weather, fatigue, or diet, the treatment is that of the intervening condition rather than of the pathological state, and exactitude, while of equal interest, is of less therapeutic importance.

